# Let’s be pragmatic about clinical data

**DOI:** 10.1038/sdata.2015.34

**Published:** 2015-07-07

Clinical research involving human participants presents challenges for any journal with policies on data sharing due to the frequent presence of personally identifiable information in clinical datasets. These challenges are particularly acute for *Scientific Data,* given our mandate that data associated with our articles must be shared as a condition of submission.

*Scientific Data* established, in 2014, a working group with stakeholders from a diverse set of organisations to investigate how the transparency of clinical research data can be increased while still protecting the privacy of individuals (http://go.nature.com/duzM7O). A draft set of guidelines and recommendations for how this might be achieved was subsequently developed and has now being shared for public comment^1^.

After publication, journals have traditionally required or encouraged clinical datasets to be available from the original investigators upon reasonable request. However, these systems have been shown to be ineffective^2–5^ in securing access to data for legitimate reanalysis. Coupled with this, there is much evidence of bias in the medical literature favouring positive results^6,7^, which can ultimately lead to ill-informed treatment decisions by clinicians.

Recently, more robust systems for accessing clinical research data on request have emerged, such as the Yale Open Data Access (YODA, http://yoda.yale.edu/) Project and Clinical Study Data Request (CSDR, https://www.clinicalstudydatarequest.com/). These initiatives ensure patient privacy and scientific legitimacy is protected by checking anonymity of datasets and only providing access to qualified researchers in a controlled access environment. Protection of participants is codified in Data Use Agreements (DUAs) and independent committees review requests to access data.

What may be missing from these systems, however, is the permanence, discoverability, accessibility and quality assurance through peer review, provided by journals—as well as robust, persistent links between the peer-reviewed literature and data repositories.

*Scientific Data* is already glad to consider datasets derived from clinical studies, and has a flexible policy acknowledging that such datasets cannot always be openly shared on the web (http://go.nature.com/rlZ4ny). This policy, however, leaves open substantial questions regarding where authors should deposit clinical datasets, and how peer review of such datasets should be conducted. Today, *Scientific Data* published its first article with a restricted-access data component^8^. This highlights the potential value of Data Descriptor articles for such data even if there is not yet a broad consensus on how journals should link articles to restricted data.

*Scientific Data* sees a role for further collaboration between journals, repositories, and these new data-on-request services. The draft guidelines, entitled ‘Publishing descriptions of non-public clinical datasets: guidance for researchers, repositories, editors and funding organisations’, were developed with input and feedback from a working group including representatives from several pharmaceutical companies, research funders and the YODA and CSDR initiatives.

The guidelines are potentially applicable to all journals that consider clinical research studies, not just data journals. Aspects of these guidelines are fairly non-controversial even if they do not yet reflect widespread practice. For example, recommendations are made for additional article sections that encourage authors to transparently declare how data can be accessed, and for the creation of persistent public landing pages at data repositories even for non-public datasets^9^.

Elements of these recommendations, however, would represent much more significant changes in current clinical data sharing and publishing behaviour. The guidelines, for example, express a clear opposition to unnecessary restrictions embedded in DUAs that are not designed to protect participant privacy, particularly co-authorship mandates. It is also recommended that data repositories should routinely allow journal peer-reviewers access to clinical datasets, in accord with DUAs, prior to publication.

These guidelines do not directly address the issue of motivating clinical researchers to *want* to share their data—although a survey found they are interested in sharing data but may not be certain how to^10,11^. *Scientific Data* hopes that additional clarity about how to share, publish and peer-review clinical data will help lower barriers to sharing, and help researchers receive more credit for their work.

*Scientific Data* encourages comments from the community by September 7th, 2015. The authors will submit the guidelines for formal publication in due course. Importantly, we also invite researchers interested in sharing their clinical datasets to prepare and submit Data Descriptors for consideration by *Scientific Data.*
